# Association of triglyceride–glucose index with coronary severity and mortality in patients on dialysis with coronary artery disease

**DOI:** 10.1186/s40001-023-01410-1

**Published:** 2023-10-17

**Authors:** Enmin Xie, Zixiang Ye, Yaxin Wu, Xuecheng Zhao, Yike Li, Nan Shen, Xiaochun Guo, Yanxiang Gao, Jingang Zheng

**Affiliations:** 1 Department of Cardiology, China-Japan Friendship Hospital, 2 Yinghua Dongjie, Beijing, 100029 China; 2 China-Japan Friendship Hospital (Institute of Clinical Medical Sciences), Chinese Academy of Medical Sciences, Peking Union Medical College, Beijing, China; 3 Department of Cardiology, Peking University China-Japan Friendship School of Clinical Medicine, Beijing, China; 4 Department of Cardiology, Fuwai Central China Cardiovascular Hospital, Henan, China

**Keywords:** Triglyceride–glucose index, Insulin resistance, Coronary artery disease, Dialysis, Prognosis

## Abstract

**Background:**

The triglyceride–glucose (TyG) index is validated as a reliable biomarker of insulin resistance and an independent predictor of cardiovascular prognosis. However, the prognostic value of the TyG index in patients on dialysis with coronary artery disease (CAD) remained unexplored. This study aimed to determine the association between the TyG index and CAD severity and mortality in these patients.

**Methods:**

A total of 1061 dialysis patients with CAD were enrolled in this multi-center cohort study from January 2015 to June 2021. The extent and severity of CAD were evaluated using the multivessel disease and Gensini score (GS). Patients were followed up for all-cause death and cardiovascular death.

**Results:**

The multivariable logistic regression model indicated that the TyG index was significantly associated with multivessel disease (odds ratio [OR] 1.51, 95% confidence interval [CI] 1.18–1.94, *P* = 0.001), and high GS (OR 1.33, 95% CI 1.10–1.61, *P* = 0.003). After adjusting for baseline risk factors, the hazards of all-cause death and cardiovascular death were 1.23 (95% CI 1.06–1.43, *P* = 0.007), and 1.33 (95% CI 1.11–1.59, *P* = 0.002), independent of CAD severity. Restricted cubic spline analysis identified a dose–response association between the TyG index and both CAD severity and mortality (all *P* for nonlinearity > 0.05). When modeling the TyG index as a categorical variable, these independent associations remained. Subgroup analyses did not substantially modify the results. Furthermore, incorporating the TyG index into the existing risk prediction model improved the predictive accuracy for all-cause death and cardiovascular death, as evaluated by C-statistic, continuous net reclassification improvement, and integrated discrimination improvement.

**Conclusions:**

In patients on dialysis with CAD, the TyG index was significantly associated with more severe CAD as well as mortality. These results highlight the clinical importance of the TyG index for assessing CAD severity and risk stratification in patients on dialysis with CAD.

**Supplementary Information:**

The online version contains supplementary material available at 10.1186/s40001-023-01410-1.

## Background

Patients with end-stage renal disease (ESRD) on dialysis have been reported to be one of the worst subsets in coronary artery disease (CAD) [1–3]. Although coronary angiography has been extensively utilized for evaluating the extent of coronary artery stenosis, the utilization of invasive CAD assessment in patients on dialysis is limited [4, 5]. Various factors contribute to this occurrence, including ambiguous CAD symptoms, a greater burden of comorbidities and frailty, and elevated surgical risks [2, 3]. Consequently, this impedes their access to appropriate medical assessments. Furthermore, traditional cardiovascular risk factors cannot fully account for the excess risk in patients on dialysis with CAD [2, 6], necessitating further exploration of prognostic variables reflecting additional dimensions of the disease among this particular patient population.

Metabolic derangements are prevalent in ESRD patients requiring dialysis and have been linked to cardiovascular pathophysiology [7]. Notably, unlike in the general population, conventional risk factors often exhibit an opposing impact in ESRD patients, a phenomenon known as “reverse epidemiology” [7]. For example, there is an inverse association between serum total cholesterol (TC) and cardiovascular risk and mortality [7, 8]. Similar trends are observed with triglycerides (TG) and high-density lipoprotein cholesterol (HDL-C) [9–11]. In addition, uremic toxins, such as asymmetric dimethylarginine, not only increase the risk of morbidity and mortality risks but can also modify or exacerbate the effects of these risk factors in ESRD patients on dialysis [12–14]. Therefore, understanding the role of specific metabolic disorders in this particular patient group is imperative.

Insulin resistance (IR) is a common metabolic disorder in ESRD patients requiring dialysis [15–17]. In dialysis patients, the development of IR is linked to many factors, including uremic toxins, chronic inflammation, oxidative stress, and abnormal adipokines [18, 19]. In addition, the contribution of IR to the development of atherosclerosis and the increased risk of adverse prognosis has been extensively demonstrated in the cardiovascular field [20, 21]. The triglyceride–glucose (TyG) index, calculated based on fasting plasma glucose (FPG) and TG, is an established surrogate marker of IR [22]. Mounting evidence supports the association of the TyG index with inflammatory processes, endothelial dysfunction, glucolipid metabolism disorders, thrombosis, and other factors contributing to atherosclerosis [23, 24]. Recent studies have revealed an association between the TyG index and CAD severity and cardiovascular events in CAD various clinical manifestations. [25–30]. In this context, the TyG index holds promise as a noninvasive assessment tool for CAD severity and risk stratification for patients on dialysis presenting with CAD. However, the association between the TyG index and the extent and severity of CAD, as well as clinical outcomes remained unexplored in these patients. Therefore, the present study aimed to determine the association between the TyG index and CAD severity and mortality in patients on dialysis with CAD.

## Methods

### Study population

The present study utilized data derived from the Coronary Revascularization in Patients On Dialysis in China-Retrospective (CRUISE-R) study (ClinicalTrials.gov NCT05841082). The CRUISE-R study was a multi-center, observational registry in China that aimed to investigate clinical characteristics, medical care, and prognostic factors of patients on dialysis with CAD. The CRUISE-R study retrospectively evaluated 455,617 cardiac catheterizations conducted between January 2015 and June 2021 in China. Several exclusion criteria were applied, including patients who did not receive dialysis therapy or had undergone dialysis treatment for less than 3 months (*n* = 453,421), those without any coronary stenosis exceeding 50% (*n* = 328), and individuals admitted for reasons other than coronary angiography (such as surgical interventions, valve diseases, or kidney transplants) (*n* = 87). For patients who were readmitted to the hospital, only data from their initial admission were considered for inclusion in our analysis. Subsequent readmissions were documented as adverse events, specifically as "readmission." (*n* = 532). As a result, a total of 1,249 patients with obstructive CAD on dialysis were enrolled in the CRUISE-R study. The study was conducted under the Declaration of Helsinki and received approval from the China–Japan Friendship Hospital Ethics Committee, with a waiver of informed consent.

**Fig. 1:**
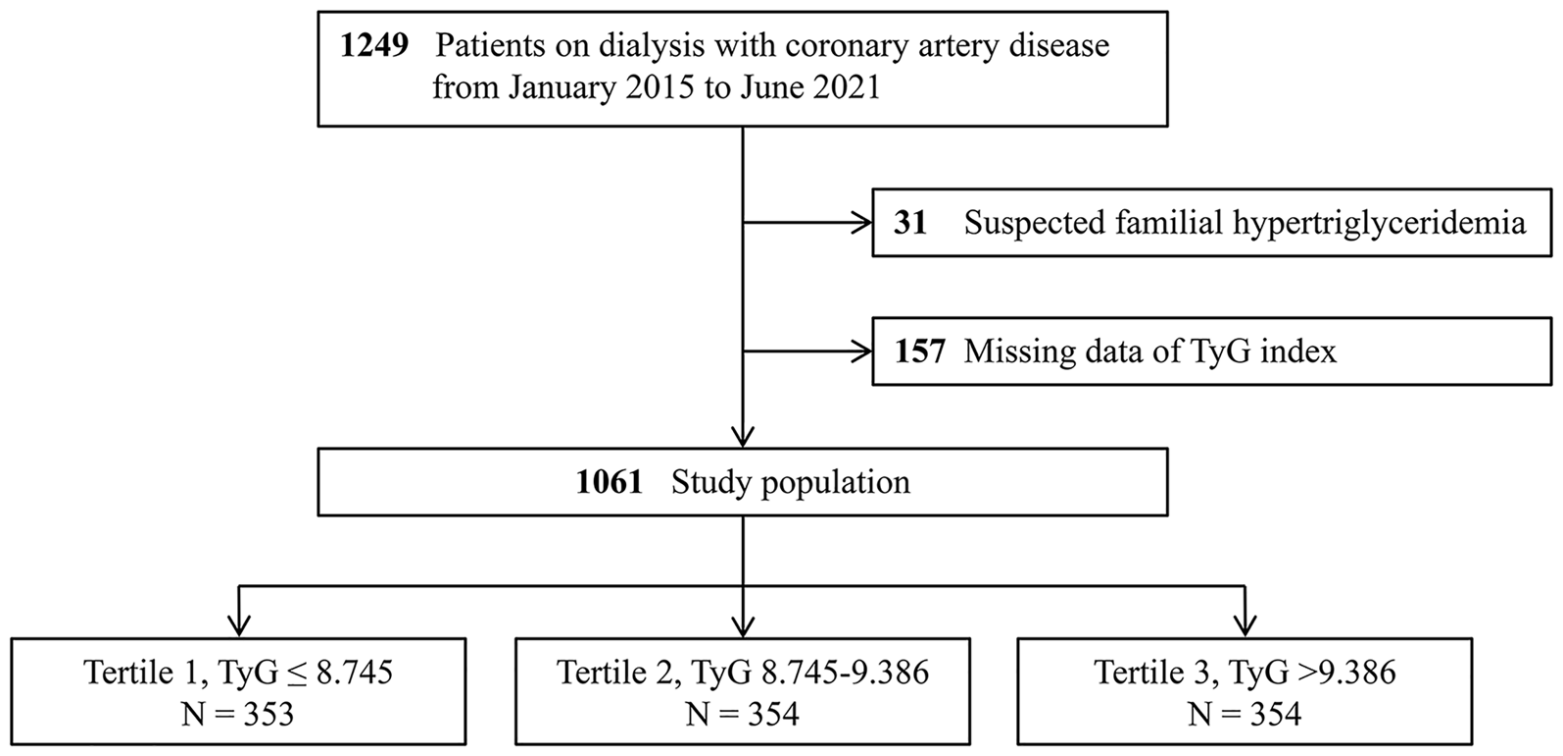
Study flowchart. *TyG* triglyceride–glucose index

For the present analysis, we further excluded 31 patients suspected of familial hypertriglyceridemia (TG ≥ 5.65 mmol/L), and 157 patients who lacked the necessary data required for TyG index calculation. Ultimately, the current analysis included 1061 participants (Fig. 1). This study followed the Strengthening the Reporting of Observational Studies in Epidemiology (STROBE) statement.

### Data collection

The data were collected from electronic medical records at each participating center by qualified study coordinators. Baseline demographic and clinical information included age, gender, systolic blood pressure, diastolic blood pressure, heart rate, hypertension, diabetes mellitus, current smokers, atrial fibrillation, cerebrovascular disease, valvular disease, peripheral arterial disease, and index presentation. Dialysis details encompassed information about dialysis modality, duration of dialysis (vintage), and dialysis cause. Laboratory measurements, such as hemoglobin, serum creatinine, TG, TC, low-density lipoprotein cholesterol (LDL-C), and HDL-C, were also obtained. The formula used to calculate the TyG index is as follows: ln [(fasting TG (mg/dl) × glucose (mg/dl)/2], using glucose and TG levels obtained within 24 h of admission [22]. In addition, details regarding coronary angiography were recorded, including the access method used, the extent of disease, the presence of moderate or severe calcification, and treatment by percutaneous coronary intervention (PCI). Furthermore, information on medication usage, such as dual antiplatelet therapy, angiotensin-converting enzyme inhibitor or angiotensin receptor blocker, *β*-blocker, calcium-channel blocker, and statin usage, was documented.

### Identification of CAD extent and severity

In this study, coronary extent and severity were assessed by the multivessel disease and Gensini score (GS) [31]. Multivessel disease is characterized by the presence of noteworthy stenosis, representing ≥ 50% of two or more main coronary arteries and/or the left main coronary artery. The GS is an assessment tool utilized in coronary angiography to evaluate the extent and severity of stenosis in coronary artery segments based on location and degree of narrowing [31]. The GS is scored as follows: 1 for 1–25% stenosis, 2 for 26–50% stenosis, 4 for 51–75% stenosis, 8 for 76–90% stenosis, 16 for 91–99% stenosis, and 32 for total occlusion. This score is then multiplied by a factor that considers the significance of the lesion’s position within the coronary arterial tree. Lesions located in the left main coronary artery were assigned a weight of 5, while the proximal left anterior descending coronary artery (LAD) or proximal left circumflex coronary artery (LCX) were assigned a weight of 2.5. Lesions located in the mid-region of the LAD were assigned a weight of 1.5, as were those located in the proximal and mid-region of the right coronary artery (RCA). Finally, lesions in the distal region of the LAD and the mid-distal regions of the LCX and RCA were assigned a weight of 1. The summation of the individual scores of each coronary artery segment produces the GS. High GS corresponds to the top tertile of scores, indicating severe coronary stenosis.

### Follow-up and outcomes

The primary outcome for follow-up was all-cause death, with the secondary outcome being cardiovascular death. Cardiovascular death was defined as death resulting from acute myocardial infarction, heart failure, sudden cardiac death, stroke, cardiovascular procedures, or cardiovascular hemorrhage. Survival and clinical assessment data were gathered by trained nurses through outpatient clinic visits and telephone interviews. The follow-up duration was extended until June 30th, 2022, and additional data related to readmitted patients were extracted from their medical records. For subjects unreachable for telephone interviews, survival status was determined based on the most recent approved timepoint, including their latest outpatient clinic visit or the last day of any hospital admission.

### Statistical analysis

The patients were divided into three categories based on the tertiles of the TyG index. Mean values with standard deviation or median values with interquartile range (25th–75th percentile) were used for continuous variables and analyzed using the ANOVA test or the Kruskal–Wallis H test, as appropriate. Categorical variables were summarized as frequency and percentages and analyzed using the Chi-square test or Fisher exact test where applicable.

Univariable and multivariable logistic regression models were employed to explore the potential association of the TyG index (modeled as continuous variables and tertiles) with the presence of multivessel disease and high GS. In Model 1, no adjustments were made, while in Model 2, we incorporated age and gender as covariates. Model 3 was a comprehensive adjustment model. The candidate variables included age, sex, systolic blood pressure, diastolic blood pressure, heart rate, hypertension, diabetes mellitus, current smoker, atrial fibrillation, cerebrovascular disease, valvular disease, peripheral arterial disease, dialysis modality, dialysis vintage, cause of dialysis, insulin therapy, index presentation, hemoglobin, TC, LDL-C, and HDL-C. Confounders that were significant in the univariate model, or of clinical importance, were included in Model 3. The results of the logistic regression model were reported as the odds ratio (OR) accompanied by a corresponding 95% confidence interval (CI). Multiple imputation was employed to estimate the missing values. Cumulative survival curves were performed using Kaplan–Meier method and compared with the log-rank test. The associations between the TyG index and all-cause death and cardiovascular death were assessed using univariable and multivariable Cox proportional hazard models, with hazard ratios (HRs) and corresponding 95% CIs reported. Proportional hazards assumptions were validated using Schoenfeld residuals. The candidate variables are listed in Table 1 (except for serum creatinine), with statistically significant or clinically relevant confounders included in the multivariable Cox analysis. To avoid collinearity and potential interactions, TG and glucose, which are components of the TyG index, were not included in the multivariable regression model. The variables included in the multivariable models were assessed for multicollinearity through the examination of variance inflation factor (VIF) values, and no indication of collinearity was discovered, as the VIF values were all below 5. Furthermore, we conducted restricted cubic spline analyses to detect potential nonlinear relationships between the TyG index and the extent and severity of CAD, as well as follow-up outcomes. The restricted cubic spline model was also adjusted for confounding factors that were included in multivariable logistic and Cox regression model. Subgroup analysis considered age, gender, diabetes mellitus, smoking, dialysis modality, index presentation, PCI treatment, high GS, and multivessel. The incremental predictive performance for follow-up outcomes after introducing the TyG index to the baseline risk model with fully adjusted variables was evaluated by calculating the C statistic, continuous net reclassification improvement (NRI), and integrated discrimination improvement (IDI). Several sensitivity analyses were performed. First, we performed a complete-case analysis to assess the association of the TyG index with coronary severity and mortality by excluding all individuals with missing values. Second, we excluded patients who received insulin therapy and repeated the analyses. Considering the important role of microinflammation in the atherosclerotic processes of ESRD patients on dialysis [32, 33], we conducted an exploratory analysis in the subgroup of 631 patients with available C-reactive protein (CRP) data. We aimed to assess whether CRP, as a marker of microinflammation, could potentially elucidate the link between the TyG index and adverse cardiovascular events. Spearman correlation analysis was conducted to examine the relationship between the TyG index and CRP levels. Subsequently, we adjusted our multivariable models for coronary severity and mortality by including CRP as a covariate. For all statistical analyses, two-sided *P* values were used, with significance considered at a value of < 0.05. We utilized SPSS 23.0 (IBM SPSS 23 Inc) and R 3.6.1 (R Development Core Team, Vienna, Austria) for data analysis.

**Table 1:** Baseline demographic and clinical data of the study patients according to tertiles of triglyceride–glucose index

Characteristic	Total *N* = 1061	Tertile 1 *N* = 353	Tertile 2 *N* = 354	Tertile 3 *N* = 354	*P* value
Age, mean (SD), yrs	61.8 (10.5)	62.3 (11.1)	60.8 (10.3)	62.2 (10.1)	0.096
Male, No. (%)	789 (74.4)	271 (76.8)	258 (72.9)	260 (73.4)	0.441
SBP, mean (SD), mmHg	141.7 (25.2)	142.1 (24.5)	143.0 (25.6)	140.1 (25.5)	0.284
DBP, mean (SD), mmHg	78.9 (13.6)	79.6 (13.5)	79.6 (13.7)	77.4 (13.4)	0.048
Heart rate, mean (SD), beats/min	80.7 (14.9)	78.9 (14.0)	81.5 (16.0)	81.8 (14.4)	0.020
Medical history and risk factors, No. (%)					
Hypertension	983 (92.6)	316 (89.5)	336 (94.9)	331 (93.5)	0.017
Diabetes mellitus	559 (52.7)	130 (36.8)	178 (50.3)	251 (70.9)	< 0.001
Current smoker	193 (18.2)	70 (19.8)	60 (16.9)	63 (17.8)	0.594
Atrial fibrillation	89 (8.4)	31 (8.8)	29 (8.2)	29 (8.2)	0.948
Cerebrovascular disease	197 (18.6)	63 (17.8)	61 (17.2)	73 (20.6)	0.466
Valvular disease	34 (3.2)	14 (4.0)	12 (3.4)	8 (2.3)	0.424
Peripheral arterial disease	104 (9.8)	30 (8.5)	34 (9.6)	40 (11.3)	0.451
Dialysis modality, No. (%)					0.048
Hemodialysis	975 (91.9)	329 (93.2)	331 (93.5)	315 (89.0)	
Peritoneal dialysis	86 (8.1)	24 (6.8)	23 (6.5)	39 (11.0)	
Vintage, years					0.308
< 1	220 (20.7)	73 (20.7)	68 (19.2)	79 (22.3)	
1–5	490 (46.2)	156 (44.2)	163 (46.0)	171 (48.3)	
5–10	278 (26.2)	94 (26.6)	104 (29.4)	80 (22.6)	
≥ 10	73 (6.9)	30 (8.5)	19 (5.4)	24 (6.8)	
Cause of dialysis, No. (%)					< 0.001
Diabetes mellitus	326 (30.7)	75 (21.2)	105 (29.7)	146 (41.2)	
Hypertension	129 (12.2)	54 (15.3)	44 (12.4)	31 (8.8)	
Glomerulonephritis	259 (24.4)	115 (32.6)	74 (20.9)	70 (19.8)	
Other/unknown	347 (32.7)	109 (30.9)	131 (37.0)	107 (30.2)	
Insulin therapy, No. (%)	353 (33.3)	76 (21.5)	106 (29.9)	171 (48.3)	< 0.001
Index presentation, No. (%)					0.543
AMI	628 (59.2)	202 (57.2)	209 (59.0)	217 (61.3)	
Non-AMI	433 (40.8)	151 (42.8)	145 (41.0)	137 (38.7)	
Hemoglobin, g/L	105.2 (19.9)	104.4 (19.3)	105.5 (19.5)	105.6 (21.0)	0.651
Glucose, mmol/L	6.3 [4.8, 9.1]	4.7 [4.2, 5.7]	6.2 [5.0, 8.0]	9.9 [7.3, 13.4]	< 0.001
Serum creatinine, mg/dl	8.6 [6.7, 11.0]	8.4 [6.9, 10.5]	9.1 [7.0, 11.6]	8.1 [6.3, 10.8]	0.004
TG, mmol/L	1.6 [1.1, 2.3]	1.0 [0.8, 1.3]	1.7 [1.4, 2.1]	2.5 [1.9, 3.4]	< 0.001
TC, mmol/L	3.7 [3.1, 4.5]	3.5 [2.9, 4.0]	3.7 [3.2, 4.4]	4.0 [3.2, 4.9]	< 0.001
HDL-C, mmol/L	0.9 [0.7, 1.1]	1.0 [0.8, 1.2]	0.8 [0.7, 1.0]	0.8 [0.7, 1.0]	< 0.001
LDL-C, mmol/L	2.1 [1.6, 2.8]	2.0 [1.6, 2.5]	2.2 [1.7, 2.8]	2.2 [1.7, 3.0]	< 0.001
TyG index	9.1 [8.5, 9.5]	8.3 [8.0, 8.5]	9.1 [8.9, 9.2]	9.8 [9.6, 10.1]	< 0.001
Procedure characteristic, No. (%)					
Radial access	823 (77.6)	264 (74.8)	281 (79.4)	278 (78.5)	0.298
Any left main disease	118 (11.1)	35 (9.9)	45 (12.7)	38 (10.7)	0.477
Multivessel disease	896 (84.4)	285 (80.7)	295 (83.3)	316 (89.3)	0.006
Moderate or severe calcification	473 (44.6)	156 (44.2)	165 (46.6)	152 (42.9)	0.607
Gensini score	52.0 [31.0, 82.0]	48.0 [27.0, 76.0]	54.0 [32.0, 84.0]	54.0 [36.0, 84.0]	0.016
PCI treatment	746 (70.3)	244 (69.1)	247 (69.8)	255 (72.0)	0.673
Discharge medications, No. (%)					
Dual antiplatelet therapy	922 (86.9)	308 (87.3)	312 (88.1)	302 (85.3)	0.522
ACE inhibitor or ARB	488 (46.0)	174 (49.3)	155 (43.8)	159 (44.9)	0.300
Beta-blocker	857 (80.8)	269 (76.2)	293 (82.8)	295 (83.3)	0.028
Calcium-channel blocker	684 (64.5)	241 (68.3)	225 (63.6)	218 (61.6)	0.162
Statin	994 (93.7)	328 (92.9)	331 (93.5)	335 (94.6)	0.635

Data are presented as mean (SD) or *n* (%)

*ACE* angiotensin-converting enzyme, *AMI* acute myocardial infarction, *ARB* angiotensin receptor blocker, *DBP* diastolic blood pressure, *HDL-C* high-density lipoprotein cholesterol, *LDL-C* low-density lipoprotein cholesterol, *PCI* percutaneous coronary intervention, *SBP* systolic blood pressure, *TC* total cholesterol, *TG* triglycerides, *TyG* triglyceride–glucose

## Results

### Baseline characteristics

The current study recruited a total of 1061 patients (Fig. 1), with a mean age of 61.8 ± 10.5 years, of whom 74.4% were male. The entire cohort exhibited a median TyG index value of 9.1 (8.5–9.5). There were a total of 24 patients with missing baseline data, which accounted for 0.7% of hemoglobin (*n* = 7), 0.6% of total cholesterol (*n* = 6), 1.5% of high-density lipoprotein cholesterol (*n* = 16), and 1.4% of low-density lipoprotein cholesterol (*n* = 15). These variables were similar before and after multiple imputation (Additional file 1: Table S1). The baseline characteristics were stratified based on the TyG index tertiles (Table 1). A higher prevalence of hypertension, diabetes mellitus, diabetes as the cause of dialysis, multivessel disease, insulin therapy, and beta-blocker therapy was observed among patients in the third TyG index tertile compared to those in the first tertile. In addition, patients with a higher TyG index exhibited a higher heart rate, TC, LDL-C, and high GS, along with lower diastolic blood pressure and HDL-C.

### Association of TyG index with coronary extent and severity

**Fig. 2:**
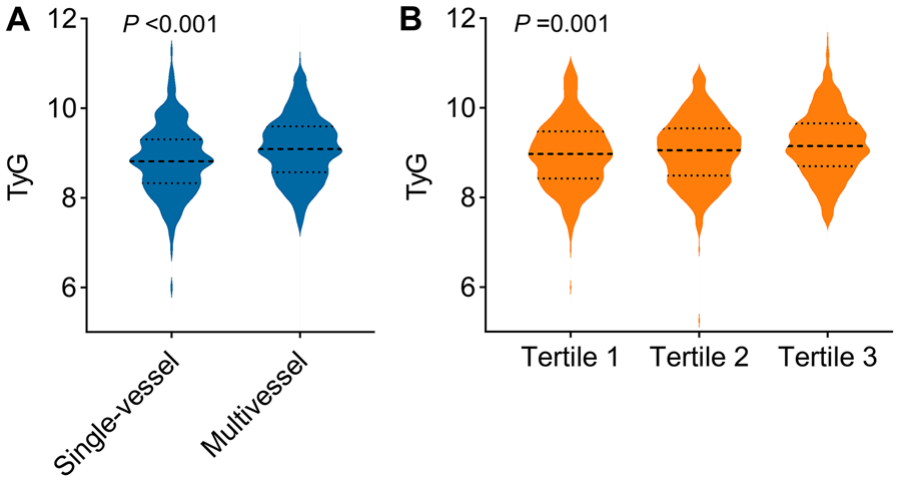
Distribution of triglyceride–glucose index according to the number of narrowed coronary arteries (**A**) and Gensini score (**B**). *TyG* triglyceride–glucose index

**Fig. 3:**
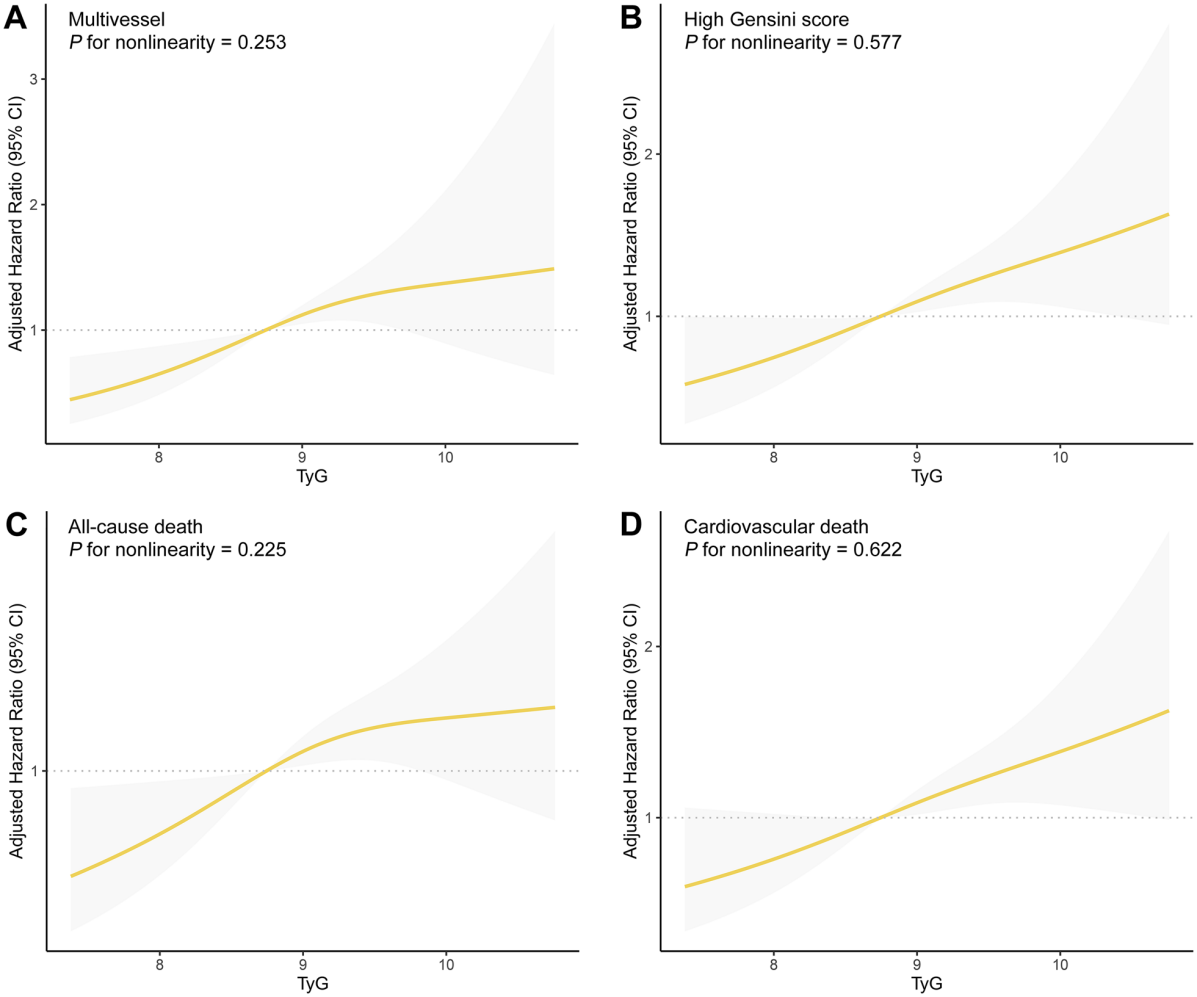
Restricted cubic spline curves of the association between triglyceride–glucose index and multivessel disease (**A**), high Gensini score (**B**), all-cause death (**C**), and cardiovascular death (**D**). Hazard ratios are indicated by solid yellow lines and 95% CIs are indicated by shaded areas. *CI* confidence interval, *TyG* triglyceride–glucose index

Patients with multivessel disease exhibited significantly higher TyG index values compared to those with single-vessel disease. (9.1 [8.6–9.6] vs 8.8 [8.3–9.3], *P* < 0.001) (Fig. 2A).In addition, as the tertile of GS score increased, the TyG index was found to gradually increase (tertile 1 [GS ≤ 38]: 9.0 [8.4, 9.5]; tertile 2 [GS 38–73]: 9.0 [8.5, 9.5]; tertile 3 [GS > 73]: 9.1 [8.7, 9.7]; *P* = 0.001) (Fig. 2B). Additional information on baseline characteristics, categorized according to the number of narrowed coronary arteries and GS, was provided in Additional file 1: Tables S2 and S3. To investigate the association between the TyG index and as high GS well as multivessel disease, logistic regression analysis was performed in this study. The univariate regression analysis (Table 2, Model 1) revealed a positive association between the TyG index as a continuous variable and high GS (OR 1.40; 95% CI 1.18–1.67). After adjustment for age and sex, the same association was observed. A subsequent full adjustment for the baseline clinical risk factors also revealed a significant elevation in the risk of high GS related to an increasing TyG index (adjusted OR, 1.33; 95% CI 1.10–1.61; *P* = 0.003) (Table 2, Model 3). Investing the tertiles of the TyG index revealed that the third tertile group had a 1.51-fold greater risk of developing high GS in the multivariate logistic regression model as compared to the reference (first) tertile group (adjusted OR 1.51; 95% CI 1.07–2.12; *P* = 0.018). In addition to high GS, the TyG index was also found to be associated with multivessel according to the univariate regression analysis (OR 1.59; 95% CI 1.27–1.99). The strength of this association remained significant after accounting for multiple possible confounders (adjusted OR 1.51; 95% CI 1.18–1.94; *P* = 0.001) (Table 2, Model 3). When comparing the highest tertile of the TyG index to the reference tertile in a fully adjusted model, a significant association with an elevated risk of multivessel was revealed (adjusted OR 1.74; 95% CI 1.09–2.77; *P* = 0.021). In addition, the findings of the restricted cubic splines are depicted in Fig. 3. The results revealed a discernible dose–response connection between the TyG index and the likelihood of high GS as well as multivessel disease, following thorough adjustment (both tests for nonlinearity revealed *P* > 0.05).

**Table 2:** Associations between triglyceride–glucose index and high Gensini score and multivessel disease

	Model 1			Model 2			Model 3		
	OR	95% CI	*P* value	OR	95% CI	*P* value	OR	95% CI	*P* value
High GS^a^									
TyG index	1.40	1.18–1.67	< 0.001	1.41	1.18–1.68	< 0.001	1.33	1.10–1.61	0.003
Tertile 1	Reference			Reference			Reference		
Tertile 2	1.37	1.00–1.89	0.053	1.39	1.00–1.92	0.047	1.32	0.94–1.85	0.105
Tertile 3	1.67	1.22–2.29	0.002	1.69	1.23–2.32	0.001	1.51	1.07–2.12	0.018
Multivessel disease									
TyG index	1.59	1.27–1.99	< 0.001	1.62	1.29–2.02	< 0.001	1.51	1.18–1.94	0.001
Tertile 1	Reference			Reference			Reference		
Tertile 2	1.19	0.81–1.75	0.369	1.24	0.84–1.83	0.278	1.13	0.75–1.70	0.564
Tertile 3	1.98	1.29–3.04	0.002	2.03	1.32–3.12	0.001	1.74	1.09–2.77	0.021

Model 1: unadjusted. Model 2: adjusted for age and sex. Model 3 for high Gensini score: adjusted for age, sex, systolic blood pressure, diastolic blood pressure, acute myocardial infarction as index presentation, insulin therapy, and high-density lipoprotein cholesterol. Model 3 for multivessel disease: adjusted for age, sex, diastolic blood pressure, acute myocardial infarction as index presentation, diabetes mellitus, cause of dialysis, insulin therapy, and high-density lipoprotein cholesterol

*CI* confidence interval, *GS* Gensini score, *OR* odds ratio, *TyG* triglyceride–glucose

^a^High GS was defined as the top Gensini score tertile

### Effect of TyG index on risk of follow-up outcomes

**Fig. 4:**
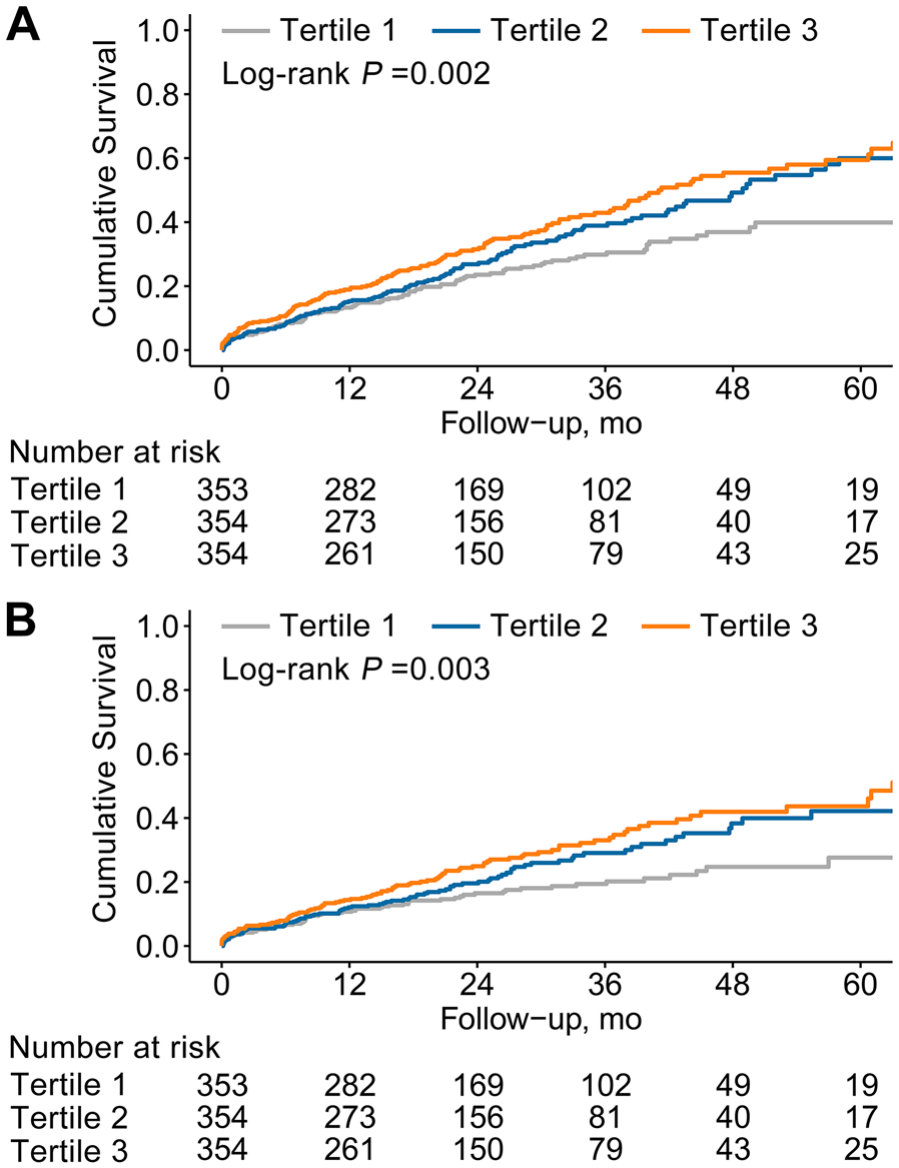
Kaplan–Meier estimated event rates of all-cause death (**A**) and cardiovascular death (**B**) according to tertiles of the triglyceride–glucose index

**Fig. 5:**
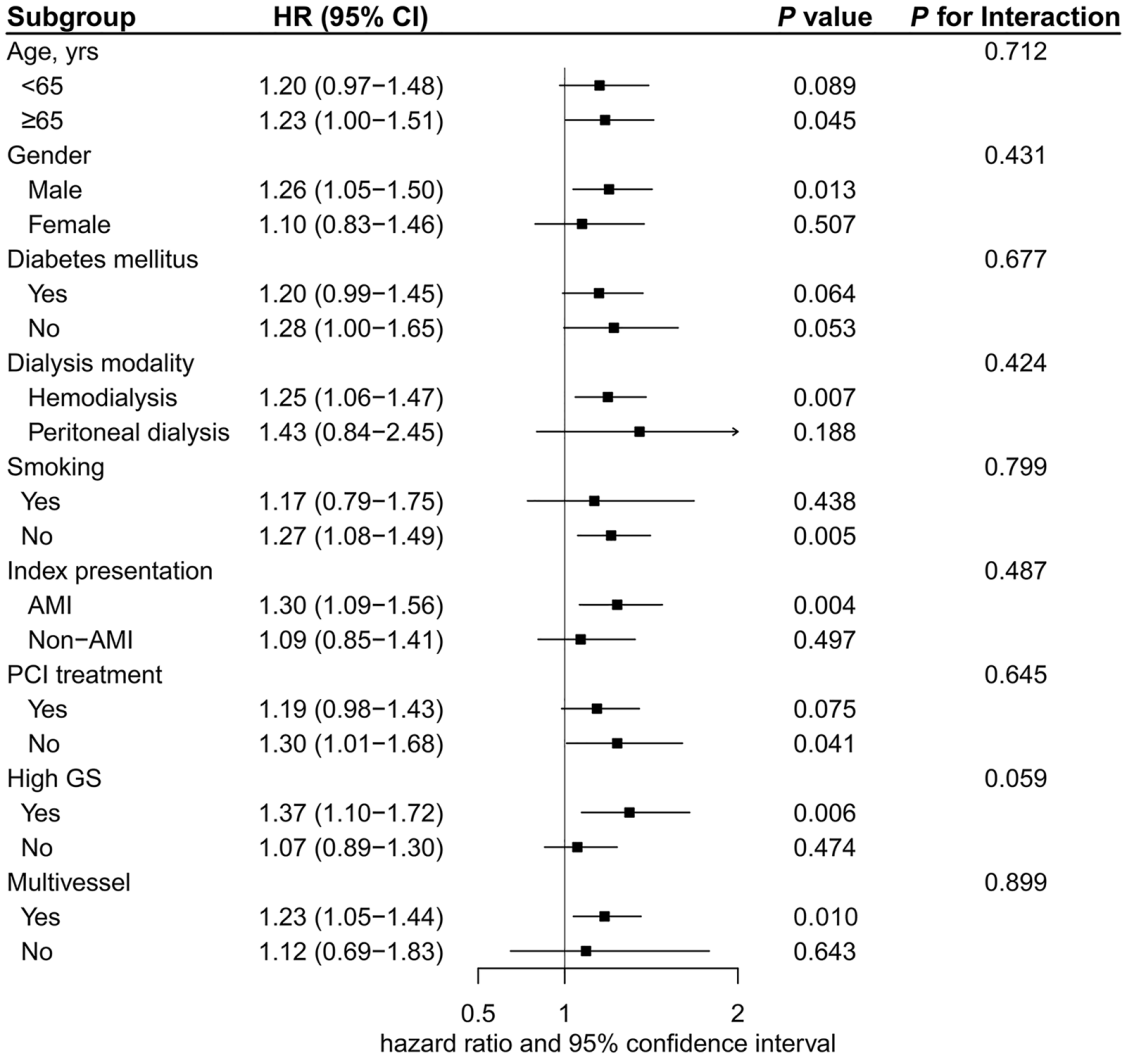
Subgroup analyses for all-cause death. *AMI* acute myocardial infarction, *CI* confidence interval, *GS* Gensini score, *HR* hazard ratio, *PCI* percutaneous coronary intervention

**Fig. 6:**
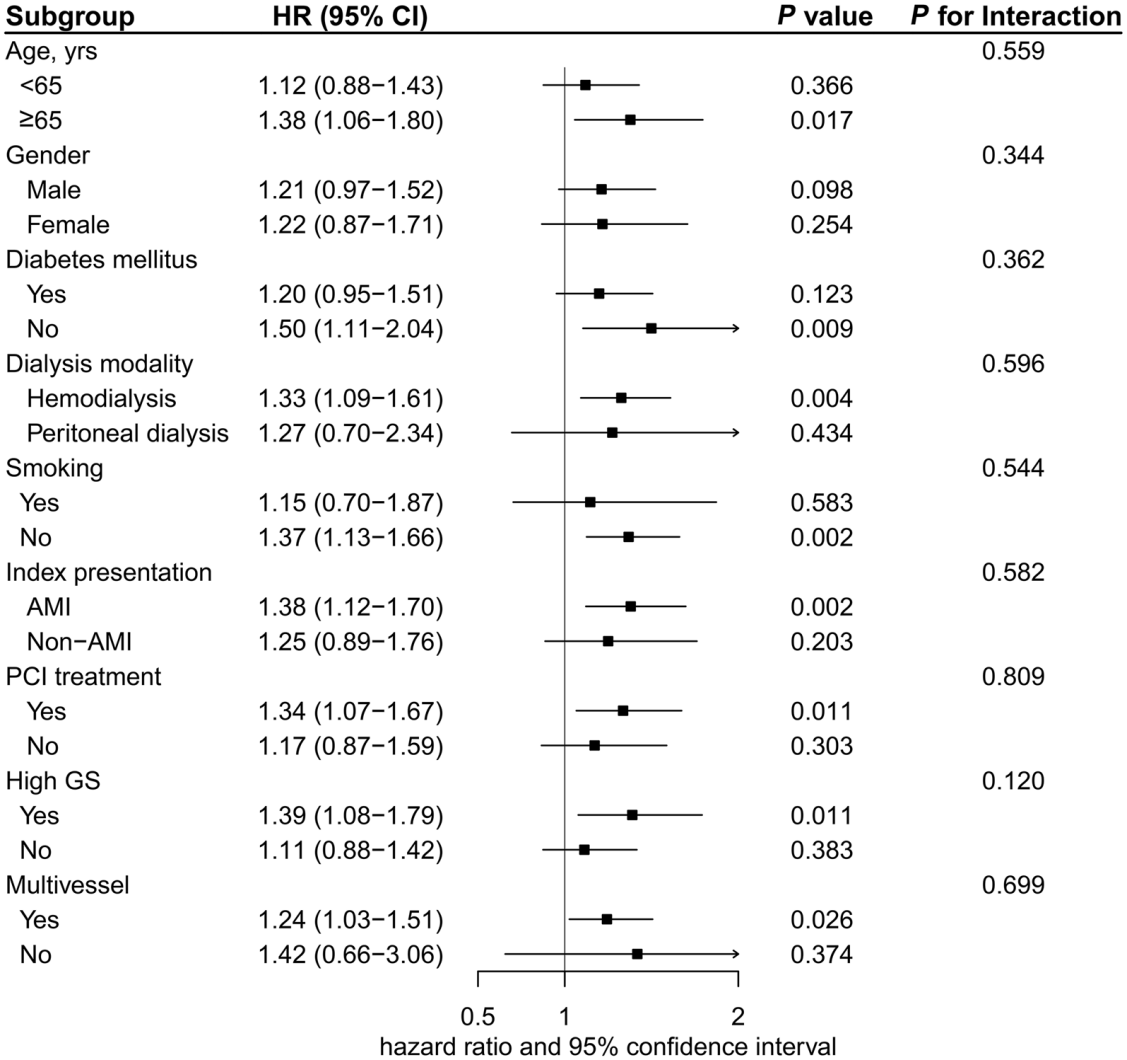
Subgroup analyses for cardiovascular death. *AMI* acute myocardial infarction, *CI* confidence interval, *GS* Gensini score, *HR* hazard ratio, *PCI* percutaneous coronary intervention

The median follow-up duration was 21.9 months (12.6–35.6 months). A total of 358 (33.7%) cases of all-cause mortality were observed, with 248 attributed to cardiovascular-related causes. Additional information on baseline characteristics between non-survivors and survivors was provided in Additional file 1: Table S4. The incidence of all-cause death was found to gradually increase as the tertile of TyG index increased: 27.2% (96/353), 34.2 (121/354), and 39.8% (141/354), respectively. Furthermore, the occurrence of cardiovascular death among those with first TyG index tertile, second TyG index tertile, and third TyG index tertile was noted as 17.8% (63/353), 23.7% (84/354), and 28.5% (101/354), respectively. Kaplan–Meier curves exhibited a significantly higher risk of both all-cause death and cardiovascular death in patients with the third TyG tertile group compared to other groups (log-rank test *P* = 0.002 and 0.003, respectively) (Fig. 4). The association between TyG index and outcomes is summarized in Table 3. When modeling TyG index as a continuous variable, a significantly increased risk of both all-cause death (adjusted HR 1.23, 95% CI 1.06–1.43, *P* = 0.007) and cardiovascular death (adjusted HR 1.33, 95% CI 1.11–1.59, *P* = 0.002) was observed in patients with high TyG index after adjustment for potential clinical risk factors (including Gensini score and multivessel). When comparing the third TyG index tertile to the first TyG index tertile, a significantly increased risk of all-cause death and cardiovascular death was identified in the fully adjusted model (adjusted HR 1.43; 95% CI 1.08–1.89; *P* = 0.012; adjusted HR 1.68; 95% CI 1.20–2.36; *P* = 0.002, respectively). Furthermore, restricted cubic splines analysis identified a discernible dose–response connection between the TyG index and the risk of all-cause death (*P* for nonlinearity = 0.225; Fig. 3C) as well as cardiovascular death (*P* for nonlinearity = 0.622; Fig. 3D). Clinically relevant variables, including age, gender, diabetes mellitus, smoking, dialysis modality, index presentation, PCI treatment, high GS, and multivessel, were used for subgroup analyses (with the TyG index modeled as a continuous variable). Despite variations in effect size and statistical significance, no significant interactions were identified regarding the risk of all-cause death in the selected subgroups (all *P* values for interaction > 0.05) (Fig. 5). Similarly, these clinically relevant variables showed no significant interaction with the TyG index regarding the risk of cardiovascular death (all *P* values for interaction > 0.05) (Fig. 6). Furthermore, the risk prediction for all-cause death was increased by adding the TyG index to the baseline risk model with fully adjusted variables (Table 4), with the C-statistic increasing from 0.698 to 0.700 (*P* = 0.007). Similarly, adding the TyG index into the baseline risk model significantly improved the predictive performance for cardiovascular death, with the C-statistic increasing from 0.725 to 0.729 (*P* = 0.005). Moreover, the NRI values for all-cause death and cardiovascular death were 0.095 (95% CI 0.009–0.170; *P* < 0.001) and 0.091 (95% CI 0.004–0.178; *P* < 0.001), respectively. Similarly, improvements were also observed using the IDI metric, with values of 0.006 (95% CI 0.001–0.021; *P* < 0.001) for all-cause death, and 0.012 (95% CI 0.001–0.029; *P* < 0.001) for cardiovascular death.

**Table 3:** Associations between triglyceride–glucose index and all-cause death and cardiovascular death

	Model 4			Model 5			Model 6		
	HR	95% CI	*P* value	HR	95% CI	*P* value	HR	95% CI	*P* value
All-cause death									
TyG index	1.32	1.15–1.52	< 0.001	1.34	1.17–1.54	< 0.001	1.23	1.06–1.43	0.007
Tertile 1	Reference			Reference				Reference	
Tertile 2	1.34	1.02–1.75	0.033	1.46	1.12–1.92	0.006	1.36	1.03–1.80	0.031
Tertile 3	1.60	1.23–2.07	< 0.001	1.61	1.24–2.08	< 0.001	1.43	1.08–1.89	0.012
Cardiovascular death									
TyG index	1.40	1.18–1.65	< 0.001	1.41	1.20–1.67	< 0.001	1.33	1.11–1.59	0.002
Tertile 1	Reference			Reference				Reference	
Tertile 2	1.41	1.02–1.95	0.040	1.53	1.01–2.12	0.011	1.52	1.08–2.14	0.016
Tertile 3	1.73	1.26–2.37	< 0.001	1.74	1.27–2.39	< 0.001	1.68	1.20–2.36	0.002

Model 4: unadjusted. Model 5: adjusted for age and sex. Model 6 for all-cause death: adjusted for age, sex, systolic blood pressure, diastolic blood pressure, heart rate, current smoker, atrial fibrillation, cerebrovascular disease, valvular disease, dialysis modality, cause of dialysis, insulin therapy, acute myocardial infarction as index presentation, left main disease, multivessel disease, moderate or severe calcification, Gensini score, angiotensin-converting enzyme inhibitor or angiotensin receptor blocker, and calcium-channel blocker. Model 6 for cardiovascular death: adjusted for age, sex, systolic blood pressure, diastolic blood pressure, diabetes mellitus, current smoker, atrial fibrillation, cause of dialysis, insulin therapy, acute myocardial infarction as index presentation, left main disease, multivessel disease, moderate or severe calcification, Gensini score, percutaneous coronary intervention treatment, angiotensin-converting enzyme inhibitor or angiotensin receptor blocker, and calcium-channel blocker

*CI* confidence interval, *HR* hazard ratio, *TyG* triglyceride–glucose

**Table 4:** Added predictive ability and reclassification statistics of triglyceride–glucose index

	C-statistic (95% CI)	*P* Value	Continuous NRI (95% CI)	*P* Value	IDI (95% CI)	*P* Value
All-cause death						
Baseline risk model^a^	0.698 (0.671, 0.725)	Reference	Reference		Reference	
Baseline risk model + TyG index	0.700 (0.673, 0.727)	0.007	0.095 (0.009, 0.170)	< 0.001	0.006 (0.001, 0.021)	< 0.001
Cardiovascular death						
Baseline risk model^a^	0.725 (0.694, 0.756)	Reference	Reference		Reference	
Baseline risk model + TyG index	0.729 (0.698, 0.760)	0.005	0.091 (0.004, 0.178)	< 0.001	0.012 (0.001, 0.029)	< 0.001

*CI* confidence interval, *IDI* integrated discrimination improvement, *NRI* net reclassification improvement, *TyG* triglyceride–glucose

^**a**^Variables included in the baseline risk model for all-cause death and cardiovascular death are shown in Model 6, Table 3

### Sensitivity analysis and exploratory analysis

Several sensitivity analyses were performed. After excluding 24 patients with missing data, we performed a complete-case analysis and identified a significant association of the TyG index with coronary severity and mortality (Additional file 1: Table S5). Furthermore, excluding 355 patients who received insulin therapy did not materially change our findings (Additional file 1: Table S6).

Given the important role of CRP-related microinflammation in the atherosclerotic processes of ESRD patients on dialysis, we conducted an exploratory analysis in the subgroup of 631 (59.5%) patients with available CRP data. The median CRP value was 9.3 mg/L (interquartile range: 3.4–25.0 mg/L). While some of the baseline variables showed significant differences between patients with and without CRP data, most of them were similar between the two groups (Additional file 1: Table S7). The Spearman correlation coefficient between the TyG index and CRP was 0.147 (*P* < 0.001). Scatter plots illustrated a positive correlation between the TyG index and CRP. After adjusting for CRP in multivariable logistic regression model (Additional file 1: Table S8), we found that the TyG index remained significantly associated with an increased risk of high Gensini score and multivessel disease, independent of CRP. These findings were consistent when examining the follow-up outcomes. In the multivariable Cox regression model (Additional file 1: Table S8), we identified a significant association between the TyG index and all-cause mortality and cardiovascular mortality, independent of CRP.

## Discussion

This multi-center cohort study identified a significant association between the TyG index and the presence of more diffuse and severe coronary artery disease (CAD), characterized by multivessel and high GS. Furthermore, a higher TyG index was significantly associated with an increased risk of all-cause death and cardiovascular death, independent of CAD severity. These correlations between the TyG index and both the severity of CAD and mortality followed a dose–response pattern. Moreover, we observed that incorporating the TyG index into the existing risk prediction model significantly improved the predictive capabilities for both all-cause death and cardiovascular death. These results highlight the significant clinical importance of TyG index for assessing CAD severity and risk stratification in CAD patients on dialysis.

IR plays a pivotal role in the pathogenesis and prognosis of several diseases, including CAD and end-stage renal disease patients requiring dialysis [15, 17, 34]. Several studies have reported an association between decreased kidney function and impaired insulin-induced glucose utilization in peripheral target tissues, leading to IR [35]. Moreover, studies have shown that IR can be detected at an early stage in patients with impaired renal function and tends to worsen as kidney function declines [18]. The etiology of IR in end-stage renal disease requiring dialysis is multifactorial and complex, which includes uremic toxins, anemia, oxidative stress, chronic inflammation, metabolic acidosis, and disturbed gut microbiome [18, 19, 36]. Furthermore, IR is a notable characteristic of metabolic syndrome and has been identified as an independent risk factor for CAD [37, 38]. The association between IR and CAD involves potential molecular mechanisms, such as endothelial dysfunction, coagulation abnormalities, impaired metabolic flexibility, and dysfunction of smooth muscle cells [37–42]. Previous research suggests that the TyG index, an accurate indicator of IR, is independently associated with coronary severity across various clinical manifestations of CAD. Su et al. documented that a higher TyG index is associated with a heightened risk of multivessel CAD in patients with CAD [43]. Moreover, another study identified a linear relationship between this correlation and its associated risk [44]. In addition, a high TyG index was found to be associated with a higher degree of complexity in coronary anatomy (as measured by the Synergy Between Percutaneous Coronary Intervention [SYNTAX] score > 22) in patients with acute coronary syndrome [27]. In this context, the TyG index has the potential to serve as a non-invasive tool to assess the complexity of CAD in dialysis patients. However, limited data exists to support the association between the TyG index and the extent and severity of CAD in this specific patient population.

Our study, based on a multi-center registry data set, provided evidence of an independent association between the TyG index and more extensive and severe CAD. In addition to the presence of multivessel disease, we employed the use of the GS to objectively quantify the severity of CAD. Despite numerically minor differences in the TyG index between groups, we found that the TyG index was significantly associated with an increased risk of multivessel disease (adjusted OR 1.51, 95% CI 1.18–1.94) and high GS (adjusted OR 1.33, 95% CI 1.10–1.61). Moreover, we observed a linear and dose–response relationship between the TyG index and the risk of developing multivessel CAD and high GS. Taken together, these results suggested that the TyG index might be a sensitive indicator of coronary severity, which is consistent with previous research in some regards [26, 27, 43, 44]. Notably, our study specifically focuses on dialysis patients with CAD, effectively bridging some gaps in understanding the associations between the TyG index and the extent and severity of CAD. Given the low acceptance of coronary angiography in patients on dialysis [4, 5], the TyG index has the potential to serve as a complementary noninvasive tool for assessing the extent and severity of CAD in this patient group.

Another important finding in this study was the independent association between the TyG index and an increased risk of all-cause death and cardiovascular death in patients on dialysis with CAD. Subgroup analyses did not identify any modifying effects on the independent association of the TyG index with mortality, including factors, such as age, diabetes, index presentation, high GS, and multivessel. Furthermore, incorporating the TyG index into the existing risk prediction model improved the predictive accuracy for all-cause death and cardiovascular death. These results align with previous research to a certain extent, supporting the prognostic value of the TyG index across various clinical manifestations of CAD. A single-center retrospective cohort study demonstrated that the TyG index was linked to an elevated risk of major adverse cardiovascular events in premature CAD, independent of traditional cardiovascular risk factors and GS [29]. Furthermore, integrating the TyG index improved outcome prediction for these patients [29]. In a nested case–control study focusing on patients with stable CAD, a positive association between the TyG index and subsequent cardiovascular events was identified [45]. Another cohort study involving individuals with acute myocardial infarction revealed a notably positive correlation between a high TyG index and both all-cause mortality and cardiovascular death over a median follow-up of 33.3 months [28]. In a large study involving 9285 patients with acute coronary syndrome, a significant relationship between the TyG index and long-term prognosis was established, regardless of diabetes status [30]. However, patients on dialysis were typically excluded or under-represented in previous studies. The significance of our current study lies in its exclusive focus on patients on dialysis with CAD, effectively expanding our understanding of the associations between the TyG index and mortality within this specific patient population. Furthermore, given the important role of CRP-related microinflammation in the atherosclerotic processes of ESRD patients on dialysis [32, 33], we conducted an exploratory analysis involving 631 patients with available CRP data. We found a significant positive correlation between TyG index and CRP. After adjusting for CRP in the multivariable models, the TyG index remained significantly associated with an increased risk of more severe CAD and mortality. These results suggest that, beyond serving as a reliable indicator of IR, the TyG index may partly reflect the microinflammation state, and thus link to adverse cardiovascular events. Overall, our study highlights the important role of the TyG index in risk stratification among patients on dialysis with CAD. This is crucial for early optimization of therapeutic regimens and focused prognosis follow-up, providing significant clinical value. Further specifically designed studies are required to determine whether interventions targeting insulin resistance, as assessed by the TyG index, have a positive impact on improving the clinical prognosis of patients on dialysis with CAD.

This study, to our knowledge, is the first to identify a significant association of the TyG index with the extent and severity of CAD as well as mortality in dialysis patients with CAD. However, our study has some limitations. First, the retrospective design of the study raised concerns regarding the influence of confounding factors and selection bias on the outcomes. Though the insulin therapy did not modify the association of the TyG index with coronary severity and mortality, information regarding oral antidiabetic agents or triglyceride-lowering drugs was not available, which might potentially influence our findings. In addition, data on other glucose-related parameters, such as the Homeostatic Model Assessment of Insulin Resistance (HOMA-IR), and insulin levels were not available. Furthermore, the TyG index was calculated using glucose and TG levels within 24 h of admission, which could be influenced by fasting status. Moreover, repeated measurements of the TyG were not performed, and advanced imaging techniques such as intravascular ultrasound or optical coherence tomography were not utilized. Future studies with larger sample sizes and more comprehensive methodologies will be necessary to validate our results and deepen our understanding of these associations.

## Conclusions

In conclusion, our study identified a positive and significant association between the TyG index and more severe CAD as well as mortality in patients on dialysis with CAD. Since the TyG index can be easily calculated through routine blood tests in clinical settings and at low cost, it has the potential to serve as a valuable biomarker for risk evaluation and stratification in this patient population. Further validation studies are necessary to confirm these findings and investigate the underlying mechanisms.

### Supplementary Information

**Additional file 1: Table S1.** Missing variables before and after multiple imputation. **Table S2.** Baseline demographic and clinical data of the study patients between single-vessel and multivessel disease. **Table S3.** Baseline demographic and clinical data of the study patients according to the Gensini score. **Table S4.** Baseline demographic and clinical data of the study patients between non-survivors and survivors. **Table S5.** Associations between triglyceride–glucose index and outcomes after excluding patients with missing data. **Table S6.** Associations between triglyceride–glucose index and outcomes after excluding patients who received insulin therapy. **Table S7.** Baseline demographic and clinical data of the study patients between patients with and without C-reaction protein data. **Table S8.** Associations between triglyceride–glucose index and outcomes in 631 patients with available C-reactive protein data. **Figure S1.** Scatter plots depicting the correlation between the triglyceride–glucose index and C-reactive protein

## Data Availability

The data set analyzed during the current study is available from the corresponding author on reasonable request.

## Abbreviations

CAD: Coronary artery disease
CI: Confidence interval
FPG: Fasting plasma glucose
GS: Gensini score
HDL-C: High-density lipoprotein cholesterol
IR: Insulin resistance
LAD: Left anterior descending
LCX: Left circumflex
LDL-C: Low-density lipoprotein cholesterol
OR: Odds ratio
RCA: Right coronary artery
TC: Total cholesterol
TG: Triglyceride
TyG: Triglyceride–glucose
