# Supplementary material for: Association of triglyceride–glucose index with coronary severity and mortality in patients on dialysis with coronary artery disease
Source: Eur J Med Res. 2023 Oct 17;28:437. doi: 10.1186/s40001-023-01410-1 (PMC10580538; doi:10.1186/s40001-023-01410-1)
Supplement: Supplementary file 1 — Additional file 1: Table S1. Missing variables before and after multiple imputation. Table S2. Baseline demographic and clinical data of the study patients between single-vessel and multivessel disease. Table S3. Baseline demographic and clinical data of the study patients according to the Gensini score. Table S4. Baseline demographic and clinical data of the study patients between non-survivors and survivors. Table S5. Associations between triglyceride–glucose index and outcomes after excluding patients with missing data. Table S6. Associations between triglyceride–glucose index and outcomes after excluding patients who received insulin therapy. Table S7. Baseline demographic and clinical data of the study patients between patients with and without C-reaction protein data. Table S8. Associations between triglyceride–glucose index and outcomes in 631 patients with available C-reactive protein data. Figure S1. Scatter plots depicting the correlation between the triglyceride–glucose index and C-reactive protein [file 40001_2023_1410_MOESM1_ESM.pdf]

Supplemental Material

Table S1. Missing variables before and after multiple imputation

Characteristic	Before imputation	After imputation	*P* value	Missing data, n (%)
Hemoglobin, g/L	105.0 ± 19.8	105.2 ± 19.9	0.820	7 (0.7)
TC, mmol/L	3.7 [3.1, 4.5]	3.7 [3.1, 4.5]	0.957	6 (0.6)
HDL-C, mmol/L	0.9 [0.7, 1.1]	0.9 [0.7, 1.1]	0.827	16 (1.5)
LDL-C, mmol/L	2.1 [1.6, 2.8]	2.1 [1.6, 2.8]	0.933	15 (1.4)

HDL-C, high-density lipoprotein cholesterol; LDL-C, low-density lipoprotein cholesterol; TC, total cholesterol.

Table S2. Baseline demographic and clinical data of the study patients between single-vessel and multivessel disease

Characteristic	Single-vessel *N* = 165	Multivessel *N* = 896	*P* value
Age, mean (SD), yrs	60.9 (11.4)	61.9 (10.3)	0.276
Male, No. (%)	111 (67.3)	678 (75.7)	0.030
SBP, mean (SD), mmHg	145.5 (28.4)	141.1 (24.6)	0.039
DBP, mean (SD), mmHg	81.2 (15.2)	78.5 (13.2)	0.019
Heart rate, mean (SD), beats/min	80.8 (15.4)	80.7 (14.8)	0.985
Medical history and risk factors, No. (%)			
Hypertension	148 (89.7)	835 (93.2)	0.156
Diabetes mellitus	72 (43.6)	487 (54.4)	0.014
Current smoker	32 (19.4)	161 (18.0)	0.744
Atrial fibrillation	17 (10.3)	72 (8.0)	0.416
Cerebrovascular disease	25 (15.2)	172 (19.2)	0.263
Valvular disease	4 (2.4)	30 (3.3)	0.705
Peripheral arterial disease	19 (11.5)	85 (9.5)	0.507
Dialysis modality, No. (%)			1.000
Hemodialysis	152 (92.1)	823 (91.9)	
Peritoneal dialysis	13 (7.9)	73 (8.1)	
Vintage, yrs			0.721
<1	36 (21.8)	184 (20.5)	
1-5	76 (46.1)	414 (46.2)	
5-10	39 (23.6)	239 (26.7)	
≥10	14 (8.5)	59 (6.6)	
Cause of dialysis, No. (%)			0.023
Diabetes mellitus	35 (21.2)	291 (32.5)	
Hypertension	24 (14.5)	105 (11.7)	
Glomerulonephritis	50 (30.3)	209 (23.3)	
Other/unknown	56 (33.9)	291 (32.5)	
Index presentation, No. (%)			<0.001
AMI	67 (40.6)	561 (62.6)	
Non-AMI	98 (59.4)	335 (37.4)	
Hemoglobin, g/L	106.8 (19.3)	104.9 (20.0)	0.255
Glucose, mmol/L	5.6 [4.6, 7.9]	6.5 [4.9, 9.4]	0.001
Serum creatinine, mg/dl	8.6 [6.7, 11.4]	8.6 [6.7, 10.8]	0.920
TG, mmol/L	1.5 [0.9, 2.0]	1.6 [1.1, 2.3]	0.002
TC, mmol/L	3.5 [3.1, 4.4]	3.7 [3.1, 4.5]	0.451
HDL-C, mmol/L	0.9 [0.8, 1.2]	0.9 [0.7, 1.1]	0.009
LDL-C, mmol/L	2.0 [1.6, 2.7]	2.1 [1.6, 2.8]	0.236

TyG index	8.8 [8.3, 9.3]	9.1 [8.6, 9.6]	<0.001
Procedure characteristic, No. (%)			
Radial access	135 (81.8)	688 (76.8)	0.186
Any left main disease	0 (0.0)	118 (13.2)	<0.001
Moderate or severe calcification	43 (26.1)	430 (48.0)	<0.001
Gensini score	20.0 [10.0, 34.0]	59.5 [39.0, 86.0]	<0.001
PCI treatment	82 (49.7)	664 (74.1)	<0.001
Discharge medications, No. (%)			
Dual antiplatelet therapy	116 (70.3)	806 (90.0)	<0.001
ACE inhibitor or ARB	72 (43.6)	416 (46.4)	0.564
Beta-blocker	120 (72.7)	737 (82.3)	0.006
Calcium-channel blocker	115 (69.7)	569 (63.5)	0.150
Statin	151 (91.5)	843 (94.1)	0.283

Data are presented as mean (SD) or n (%).

ACE, angiotensin-converting enzyme; AMI, acute myocardial infarction; ARB, angiotensin receptor blocker; DBP, diastolic blood pressure; HDL-C, high-density lipoprotein cholesterol; LDL-C, low-density lipoprotein cholesterol; PCI, percutaneous coronary intervention; SBP, systolic blood pressure; TC, total cholesterol; TG, triglycerides; TyG, triglyceride-glucose index.

Table S3. Baseline demographic and clinical data of the study patients according to the Gensini score

Characteristic	GS ≤73<br>*N* = 709	GS >73<br>*N* = 352	*P* value
Age, mean (SD), yrs	61.8 (10.5)	61.7 (10.4)	0.813
Male, No. (%)	518 (73.1)	271 (77.0)	0.192
SBP, mean (SD), mmHg	144.1 (25.1)	136.9 (25.0)	<0.001
DBP, mean (SD), mmHg	79.9 (13.6)	76.9 (13.3)	0.001
Heart rate, mean (SD), beats/min	80.2 (15.0)	81.8 (14.5)	0.119
Medical history and risk factors, No. (%)			
Hypertension	655 (92.4)	328 (93.2)	0.731
Diabetes mellitus	366 (51.6)	193 (54.8)	0.358
Current smoker	132 (18.6)	61 (17.3)	0.669
Atrial fibrillation	58 (8.2)	31 (8.8)	0.819
Cerebrovascular disease	138 (19.5)	59 (16.8)	0.326
Valvular disease	19 (2.7)	15 (4.3)	0.233
Peripheral arterial disease	76 (10.7)	28 (8.0)	0.188
Dialysis modality, No. (%)			1.000
Hemodialysis	652 (92.0)	323 (91.8)	
Peritoneal dialysis	57 (8.0)	29 (8.2)	
Vintage, yrs			0.627
<1	141 (19.9)	79 (22.4)	
1-5	337 (47.5)	153 (43.5)	
5-10	183 (25.8)	95 (27.0)	
≥10	48 (6.8)	25 (7.1)	
Cause of dialysis, No. (%)			0.593
Diabetes mellitus	214 (30.2)	112 (31.8)	
Hypertension	86 (12.1)	43 (12.2)	
Glomerulonephritis	182 (25.7)	77 (21.9)	
Other/unknown	227 (32.0)	120 (34.1)	
Index presentation, No. (%)			<0.001
AMI	378 (53.3)	250 (71.0)	
Non-AMI	331 (46.7)	102 (29.0)	
Hemoglobin, g/L	104.7 (19.9)	106.1 (19.9)	0.287
Glucose, mmol/L	6.0 [4.7, 8.7]	6.8 [5.2, 9.9]	<0.001
Serum creatinine, mg/dl	8.7 [6.7, 10.9]	8.4 [6.6, 11.0]	0.806
TG, mmol/L	1.6 [1.1, 2.2]	1.7 [1.2, 2.4]	0.042
TC, mmol/L	3.7 [3.1, 4.5]	3.7 [3.1, 4.5]	0.901
HDL-C, mmol/L	0.9 [0.8, 1.1]	0.9 [0.7, 1.0]	0.006
LDL-C, mmol/L	2.1 [1.6, 2.8]	2.1 [1.7, 2.8]	0.722

TyG index	9.0 [8.5, 9.5]	9.1 [8.7, 9.7]	<0.001
Procedure characteristic, No. (%)			
Radial access	572 (80.7)	251 (71.3)	0.001
Any left main disease	22 (3.1)	96 (27.3)	<0.001
Moderate or severe calcification	291 (41.0)	182 (51.7)	0.001
Gensini score	39.0 [23.0, 52.0]	97.0 [82.0, 120.0]	<0.001
PCI treatment	491 (69.3)	255 (72.4)	0.317
Discharge medications, No. (%)			
Dual antiplatelet therapy	598 (84.3)	324 (92.0)	0.001
ACE inhibitor or ARB	325 (45.8)	163 (46.3)	0.937
Beta-blocker	562 (79.3)	295 (83.8)	0.092
Calcium-channel blocker	485 (68.4)	199 (56.5)	<0.001
Statin	663 (93.5)	331 (94.0)	0.845

Data are presented as mean (SD) or n (%).

ACE, angiotensin-converting enzyme; AMI, acute myocardial infarction; ARB, angiotensin receptor blocker; DBP, diastolic blood pressure; GS, Gensini score; HDL-C, high-density lipoprotein cholesterol; LDL-C, low-density lipoprotein cholesterol; PCI, percutaneous coronary intervention; SBP, systolic blood pressure; TC, total cholesterol; TG, triglycerides; TyG, triglyceride-glucose index.

Table S4. Baseline demographic and clinical data of the study patients between non-survivors and survivors

Characteristic	Non-survivor<br>*N* = 703	Survivor<br>*N* = 358	*P* value
Age, mean (SD), yrs	60.1 (10.3)	65.1 (10.0)	<0.001
Male, No. (%)	529 (75.2)	260 (72.6)	0.395
SBP, mean (SD), mmHg	143.3 (25.4)	138.6 (24.7)	0.004
DBP, mean (SD), mmHg	80.1 (13.5)	76.5 (13.5)	<0.001
Heart rate, mean (SD), beats/min	79.9 (14.4)	82.4 (15.6)	0.012
Medical history and risk factors, No. (%)			
Hypertension	648 (92.2)	335 (93.6)	0.483
Diabetes mellitus	349 (49.6)	210 (58.7)	0.007
Current smoker	138 (19.6)	55 (15.4)	0.105
Atrial fibrillation	45 (6.4)	44 (12.3)	0.002
Cerebrovascular disease	117 (16.6)	80 (22.3)	0.030
Valvular disease	19 (2.7)	15 (4.2)	0.264
Peripheral arterial disease	64 (9.1)	40 (11.2)	0.336
Dialysis modality, No. (%)			0.192
Hemodialysis	652 (92.7)	323 (90.2)	
Peritoneal dialysis	51 (7.3)	35 (9.8)	
Vintage, yrs			0.269
<1	152 (21.6)	68 (19.0)	
1-5	310 (44.1)	180 (50.3)	
5-10	189 (26.9)	89 (24.9)	
≥10	52 (7.4)	21 (5.9)	
Cause of dialysis, No. (%)			0.002
Diabetes mellitus	193 (27.5)	133 (37.2)	
Hypertension	79 (11.2)	50 (14.0)	
Glomerulonephritis	185 (26.3)	74 (20.7)	
Other/unknown	246 (35.0)	101 (28.2)	
Index presentation, No. (%)			<0.001
AMI	384 (54.6)	244 (68.2)	
Non-AMI	319 (45.4)	114 (31.8)	
Hemoglobin, g/L	105.4 (20.3)	104.8 (19.2)	0.647
Glucose, mmol/L	5.8 [4.7, 8.3]	7.4 [5.4, 10.4]	<0.001
Serum creatinine, mg/dl	8.8 [6.9, 11.2]	8.0 [6.5, 10.4]	0.004
TG, mmol/L	1.6 [1.1, 2.3]	1.6 [1.1, 2.2]	0.695
TC, mmol/L	3.6 [3.1, 4.5]	3.8 [3.1, 4.5]	0.326
HDL-C, mmol/L	0.9 [0.7, 1.1]	0.9 [0.7, 1.1]	0.692
LDL-C, mmol/L	2.1 [1.6, 2.8]	2.2 [1.7, 2.8]	0.386

TyG index	9.0 [8.5, 9.5]	9.2 [8.7, 9.6]	<0.001
Procedure characteristic, No. (%)			
Radial access	560 (79.7)	263 (73.5)	0.027
Any left main disease	61 (8.7)	57 (15.9)	0.001
Moderate or severe calcification	294 (41.8)	179 (50.0)	0.014
Multivessel	568 (80.8)	328 (91.6)	<0.001
Gensini score	48.0 [28.0, 77.5]	62.8 [39.0, 90.0]	<0.001
PCI treatment	508 (72.3)	238 (66.5)	0.060
Discharge medications, No. (%)			
Dual antiplatelet therapy	601 (85.5)	321 (89.7)	0.070
ACE inhibitor or ARB	341 (48.5)	147 (41.1)	0.025
Beta-blocker	572 (81.4)	285 (79.6)	0.546
Calcium-channel blocker	470 (66.9)	214 (59.8)	0.027
Statin	665 (94.6)	329 (91.9)	0.116

Data are presented as mean (SD) or n (%).

ACE, angiotensin-converting enzyme; AMI, acute myocardial infarction; ARB, angiotensin receptor blocker; DBP, diastolic blood pressure; HDL-C, high-density lipoprotein cholesterol; LDL-C, low-density lipoprotein cholesterol; PCI, percutaneous coronary intervention; SBP, systolic blood pressure; TC, total cholesterol; TG, triglycerides; TyG, triglyceride-glucose index.

Table S5. Associations between triglyceride-glucose index and outcomes after excluding patients with missing data

Characteristic	Univariate			Multivariable		
	OR or HR	95% CI	*P* value	OR or HR	95% CI	*P* value
High GS[a]	1.38	1.15-1.64	<0.001	1.31	1.09-1.58	0.004
Multivessel disease	1.65	1.31-2.07	<0.001	1.54	1.19-1.98	0.001
All-cause death	1.31	1.14-1.51	<0.001	1.22	1.05-1.43	0.009
Cardiovascular death	1.38	1.17-1.64	<0.001	1.33	1.10-1.60	0.003

[a] High GS was defined as the top Gensini score tertile.

CI, confidence interval; GS, Gensini score; HR, hazard ratio; OR, odds ratio.

Table S6. Associations between triglyceride-glucose index and outcomes after excluding patients received insulin therapy

Characteristic	Univariate			Multivariable		
	**OR or HR**	**95% CI**	***P* value**	**OR or HR**	**95% CI**	***P* value**
High GS[a]	1.57	1.24-1.98	<0.001	1.44	1.12-1.85	0.004
Multivessel disease	2.01	1.50-2.70	<0.001	1.91	1.39-2.62	<0.001
All-cause death	1.41	1.17-1.70	<0.001	1.33	1.09-1.62	0.006
Cardiovascular death	1.62	1.28-2.05	<0.001	1.49	1.18-1.88	0.001

[a] High GS was defined as the top Gensini score tertile.

CI, confidence interval; GS, Gensini score; HR, hazard ratio; OR, odds ratio.

Table S7. Baseline demographic and clinical data of the study patients between patients with and without C-reaction protein data

**Characteristic**	**Patients with CRP data *N* = 631**	**Patients without CRP data *N* = 430**	***P* value**
Age, mean (SD), yrs	61.7 (10.3)	61.8 (10.7)	0.913
Male, No. (%)	487 (77.2)	302 (70.2)	0.013
SBP, mean (SD), mmHg	142.2 (24.6)	141.0 (26.2)	0.440
DBP, mean (SD), mmHg	78.5 (12.9)	79.5 (14.5)	0.218
Heart rate, mean (SD), beats/min	81.1 (15.2)	80.3 (14.3)	0.393
Medical history and risk factors, No. (%)			
Hypertension	586 (92.9)	397 (92.3)	0.831
Diabetes mellitus	346 (54.8)	213 (49.5)	0.102
Current smoker	120 (19.0)	73 (17.0)	0.444
Atrial fibrillation	56 (8.9)	33 (7.7)	0.562
Cerebrovascular disease	131 (20.8)	66 (15.3)	0.032
Valvular disease	22 (3.5)	12 (2.8)	0.650
Peripheral arterial disease	65 (10.3)	39 (9.1)	0.577
Dialysis modality, No. (%)			0.403
Hemodialysis	584 (92.6)	391 (90.9)	
Peritoneal dialysis	47 (7.4)	39 (9.1)	
Vintage, yrs			0.641
<1	130 (20.6)	90 (20.9)	
1-5	299 (47.4)	191 (44.4)	
5-10	157 (24.9)	121 (28.1)	
≥10	45 (7.1)	28 (6.5)	
Cause of dialysis, No. (%)			<0.001
Diabetes mellitus	209 (33.1)	117 (27.2)	
Hypertension	78 (12.4)	51 (11.9)	
Glomerulonephritis	177 (28.1)	82 (19.1)	
Other/unknown	167 (26.5)	180 (41.9)	
Index presentation, No. (%)			0.999
AMI	374 (59.3)	254 (59.1)	
Non-AMI	257 (40.7)	176 (40.9)	
Hemoglobin, g/L	104.3 (20.3)	106.4 (19.3)	0.097
Glucose, mmol/L	6.1 [4.8, 9.1]	6.5 [4.8, 9.0]	0.352
Serum creatinine, mg/dl	8.8 [6.6, 11.1]	8.4 [6.7, 10.7]	0.276
TG, mmol/L	1.5 [1.1, 2.2]	1.7 [1.2, 2.4]	0.003
TC, mmol/L	3.7 [3.1, 4.4]	3.7 [3.1, 4.6]	0.351
HDL-C, mmol/L	0.9 [0.7, 1.1]	0.9 [0.7, 1.1]	0.324

LDL-C, mmol/L	2.1 [1.7, 2.7]	2.1 [1.6, 2.8]	0.847
Procedure characteristic, No. (%)			
Radial access	471 (74.6)	352 (81.9)	0.007
Any left main disease	66 (10.5)	52 (12.1)	0.465
Moderate or severe calcification	278 (44.1)	195 (45.3)	0.724
Multivessel	536 (84.9)	360 (83.7)	0.650
Gensini score	52.0 [32.0, 78.5]	54.0 [31.0, 86.0]	0.125
PCI treatment	448 (71.0)	298 (69.3)	0.599
Discharge medications, No. (%)			
Dual antiplatelet therapy	539 (85.4)	383 (89.1)	0.102
ACE inhibitor or ARB	313 (49.6)	175 (40.7)	0.005
Beta-blocker	513 (81.3)	344 (80.0)	0.654
Calcium-channel blocker	401 (63.5)	283 (65.8)	0.489
Statin	598 (94.8)	396 (92.1)	0.103

Data are presented as mean (SD) or n (%).

ACE, angiotensin-converting enzyme; AMI, acute myocardial infarction; ARB, angiotensin receptor blocker; CRP, C-reaction protein; DBP, diastolic blood pressure; HDL-C, high-density lipoprotein cholesterol; LDL-C, low-density lipoprotein cholesterol; PCI, percutaneous coronary intervention; SBP, systolic blood pressure; TC, total cholesterol; TG, triglycerides.

Table S8. Associations between triglyceride-glucose index and outcomes in 631 patients with available C-reactive protein data

Characteristic	High GS[a]		Multivessel disease		All-cause death		Cardiovascular death	
	OR/HR (95% CI)	*P* value	OR/HR (95% CI)	*P* value	OR/HR (95% CI)	*P* value	OR/HR (95% CI)	*P* value
Univariate								
TyG index	1.683 (1.331-2.130)	<0.001	1.650 (1.224-2.225)	0.001	1.368 (1.147-1.632)	<0.001	1.496 (1.210-1.851)	<0.001
CRP	1.005 (1.001-1.009)	0.009	1.007 (0.999-1.014)	0.075	1.006 (1.003-1.008)	<0.001	1.006 (1.003-1.009)	<0.001
Multivariable								
TyG index	1.555 (1.193-2.026)	0.001	1.471 (1.065-2.030)	0.019	1.242 (1.022-1.510)	0.029	1.423 (1.126-1.799)	0.003
CRP	1.002 (0.997-1.006)	0.471	1.003 (0.996-1.011)	0.365	1.004 (1.001-1.006)	0.017	1.003 (0.999-1.006)	0.124

[a] High GS was defined as the top Gensini score tertile.

CI, confidence interval; CRP, C-reactive protein; GS, Gensini score; HR, hazard ratio; OR, odds ratio.

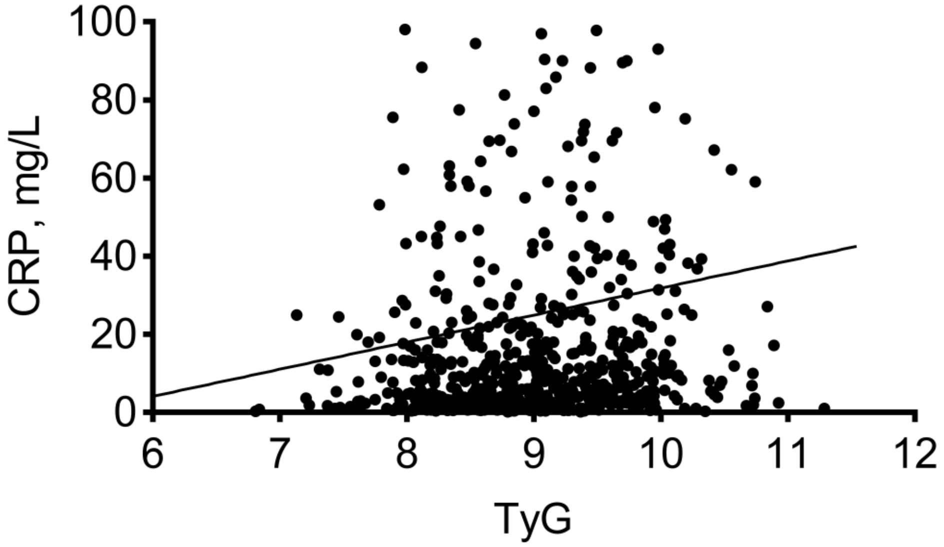

Figure S1. Scatter plots depicting the correlation between the triglyceride-glucose index and C-reactive protein
